# Supplementary material for: Activation of GCN2/ATF4 signals in amygdalar PKC-δ neurons promotes WAT browning under leucine deprivation
Source: Nat Commun. 2020 Jun 5;11:2847. doi: 10.1038/s41467-020-16662-2 (PMC7275074; doi:10.1038/s41467-020-16662-2)
Supplement: Supplementary file 1 — Supplementary Information [file 41467_2020_16662_MOESM1_ESM.pdf]

Activation of GCN2/ATF4 signals in amygdalar PKC-δ neurons promotes WAT browning under leucine deprivation

Feixiang Yuan[1], Haizhou Jiang[1], Hanrui Yin[1], Xiaoxue Jiang[1], Fuxin Jiao[1], Shanghai Chen[1], Hao Ying[1], Yan Chen[1], Qiwei Zhai[1] and Feifan Guo[1#]

[1]CAS Key Laboratory of Nutrition, Metabolism and Food safety, Shanghai Institute of Nutrition and Health, Shanghai Institutes for Biological Sciences, University of Chinese Academy of Sciences, Chinese Academy of Sciences

[#]Correspondence should be addressed to Feifan Guo; E-mail: ffguo@sibs.ac.cn; Address: 320 Yueyang Road, Shanghai, China 200031

Supplementary Figure Legends and Tables

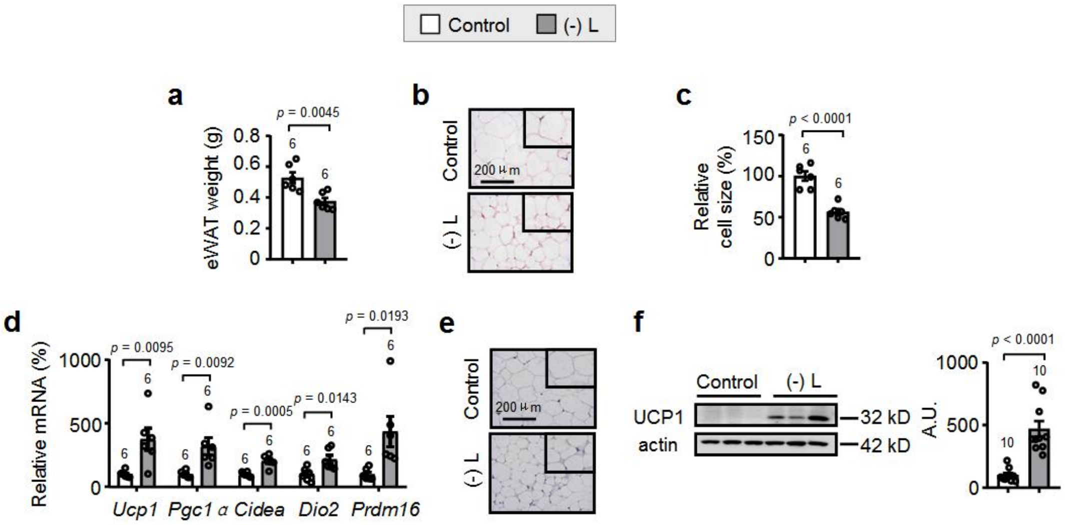

Supplementary Figure 1. Metabolic parameters related to leucine deprivation. a Epididymal WAT (eWAT) weight. **b** Representative images of hematoxylin and eosin (H&E) staining of eWAT. **c** eWAT cell size quantified by Image J analysis of H&E images. **d** Gene expression of *Ucp1, Pgc1a, Cidea, Dio2* and *Prdm16* in eWAT by RT-PCR. **e** Representative images of immunohistochemistry (IHC) of UCP1 in eWAT. **f** UCP1 protein in eWAT by western blotting (left) and quantified by densitometric analysis (right), A.U.: arbitrary units. Studies were conducted using 14- to 15-week-old male wild-type mice fed a control (Control) or leucine-deficient [(-) L] diet for 3 days. Data are expressed as the mean ± SEM (n represents number of samples and are indicated above the bar graph), with individual data points. Data were analyzed by two-tailed unpaired Student's t test. Source data are provided as a Source Data file.

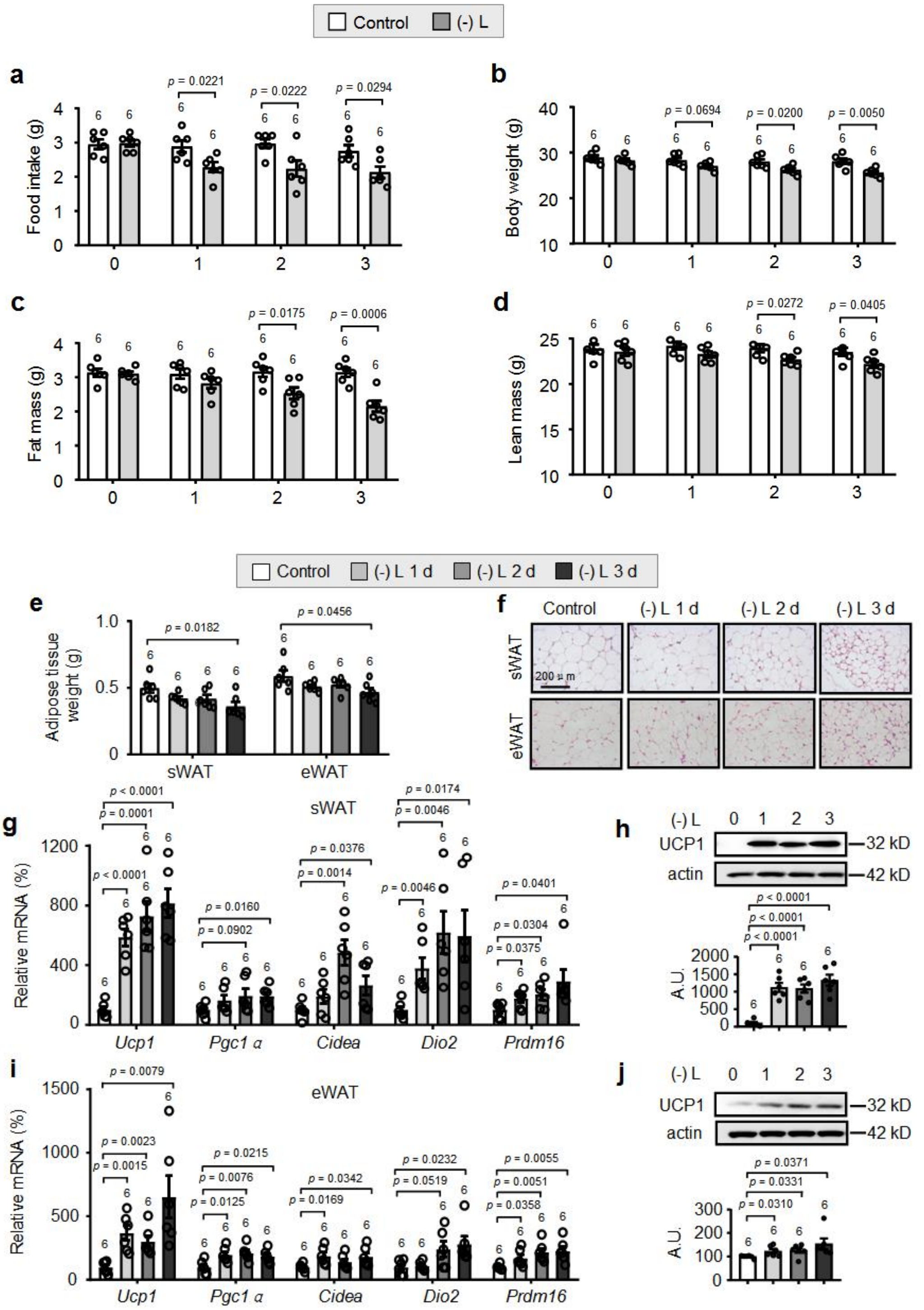

Supplementary Figure 2. Metabolic parameters over the three days period under leucine deprivation. a Food intake. **b** Body weight. **c** Fat mass by NMR. **d** Lean mass by NMR. **e** Subcutaneous WAT (sWAT) and Epididymal WAT (eWAT) weight. **f** Representative images of hematoxylin and eosin (H&E) staining of sWAT and

eWAT. **g** Gene expression of *Ucp1, Pgc1a, Cidea, Dio2* and *Prdm16* in sWAT by RT-PCR. **h** UCP1 protein in sWAT by western blotting (top) and quantified by densitometric analysis (bottom), A.U.: arbitrary units. **i** Gene expression of *Ucp1, Pgc1a, Cidea, Dio2* and *Prdm16* in eWAT by RT-PCR. **j** UCP1 protein in eWAT by western blotting (top) and quantified by densitometric analysis (bottom). Studies were conducted using 16- to 17-week-old male wild-type mice fed a control (Control) or leucine-deficient [(-) L] diet for three days for **a-d**, or provided with a leucine-deficient diet one day [(-) L 1 d], two days [(-) L 2 d], three days [(-) L 3 d] or without this diet (Control) prior to be used for **e-j**. Data are expressed as the mean ± SEM (n represents number of samples and are indicated above the bar graph), with individual data points. Data were analyzed by two-tailed unpaired Student's t test for **a-d**, or by one-way ANOVA followed by the SNK (Student–Newman–Keuls) test for **e-j**. Source data are provided as a Source Data file.

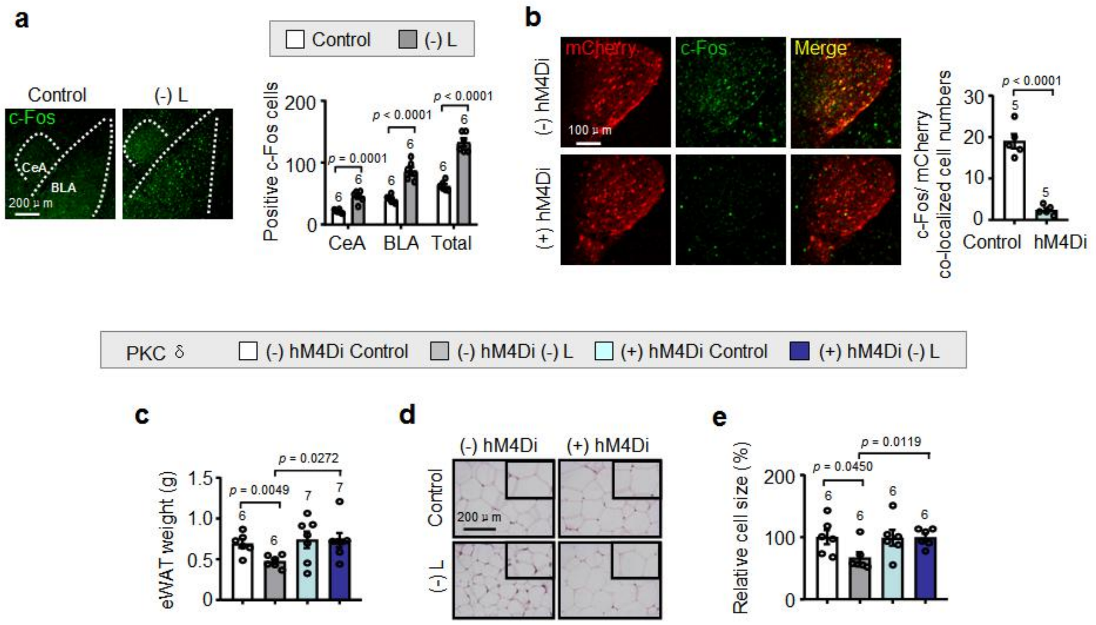

Supplementary Figure 3. Metabolic parameters related to mice with inhibition of PKC-δ neuronal activity under leucine deprivation. a Immunofluorescence (IF) staining for c-Fos (green) in amygdala sections and quantification of c-Fos positive cells. CeA: the central nuclei of the amygdala; BLA: the basolateral nuclei of the amygdala. **b** IF staining for mCherry (red), c-Fos (green) and merge (yellow) in CeA sections (left), and quantification of c-Fos and mCherry colocalized cell numbers (right). **c** Epididymal WAT (eWAT) weight. **d** Representative images of hematoxylin and eosin (H&E) staining of eWAT. **e** eWAT cell size quantified by Image J analysis of H&E images. Studies for **a** were conducted using 14- to 15-week-old male wild-type mice fed a control (Control) or leucine-deficient [(-) L] diet for 3 days; studies for **b**-**e** were conducted using 22- to 24-week-old male PKC-δ-Cre mice receiving AAVs expressing mCherry (PKCδ - hM4Di) or hM4Di (PKCδ + hM4Di) and fed a Control or (-) L diet for 3 days. Data are expressed as the mean ± SEM (n represents number of samples and are indicated above the bar graph), with individual data points. Data were analyzed by one-way ANOVA followed by the SNK (Student–Newman–Keuls) test for **c** and **e**, or by two-tailed unpaired Student's t test for **a** and **b**. Source data are provided as a Source Data file.

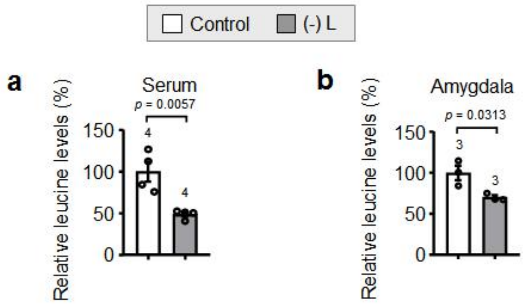

Supplementary Figure 4. Leucine deprivation decreases leucine levels in serum and amygdala. a Leucine levels in serum. **b** Leucine levels in the amygdala. Studies were conducted using 14- to 15-week-old male wild-type mice fed a control (Control) or leucine-deficient [(-) L] diet for 3 days. Data are expressed as the mean ± SEM (n represents number of samples and are indicated above the bar graph), with individual data points. Data were analyzed by two-tailed unpaired Student's t test. Source data are provided as a Source Data file.

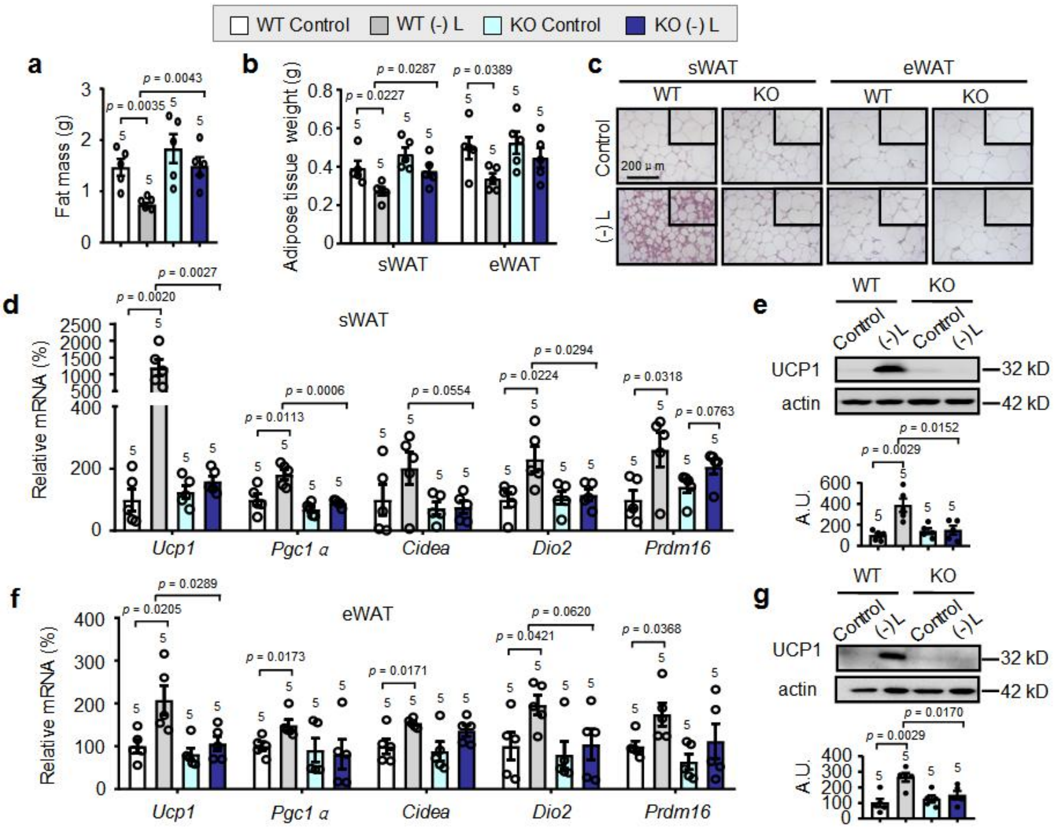

Supplementary Figure 5. Leucine deprivation-stimulated WAT browning is blocked in GCN2$^{-/-}$ mice. **a** Fat mass by NMR. **b** Subcutaneous WAT (sWAT) and epididymal WAT (eWAT) weight. **c** Representative images of hematoxylin and eosin (H&E) staining of sWAT and eWAT. **d** Gene expression of *Ucp1, Pgc1a, Cidea, Dio2* and *Prdm16* in sWAT by RT-PCR. **e** UCP1 protein in sWAT by western blotting (top) and quantified by densitometric analysis (bottom), A.U.: arbitrary units. **f** Gene expression of *Ucp1, Pgc1a, Cidea, Dio2* and *Prdm16* in eWAT by RT-PCR. **g** UCP1 protein in eWAT by western blotting (top) and quantified by densitometric analysis (bottom). Studies were conducted using 9- to 10-week-old male wild-type (WT) or GCN2$^{-/-}$ (KO) mice fed a control (Control) or leucine-deficient [(-) L] diet for 3 days. Data are expressed as the mean ± SEM (n represents number of samples and are indicated above the bar graph), with individual data points. Data were analyzed by

one-way ANOVA followed by the SNK (Student–Newman–Keuls) test. Source data are provided as a Source Data file.

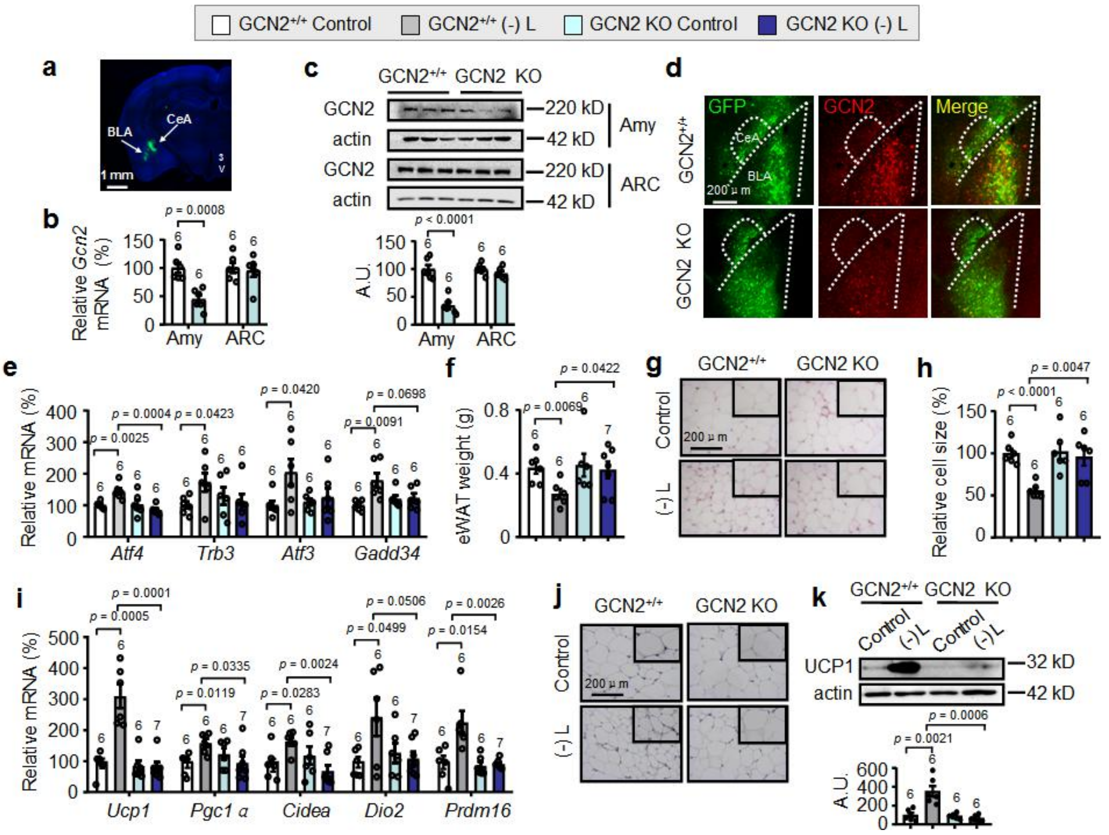

Supplementary Figure 6. Metabolic parameters in mice with GCN2 deletion in amygdala (GCN2 KO) under leucine deprivation. a Post hoc visualization of GFP (green) and DAPI (blue) in the amygdala in a $GCN2^{+/+}$ mice receiving AAV-Cre-GFP stereotaxic injections (n=3); CeA: the central nuclei of the amygdala; BLA: the basolateral nuclei of the amygdala; 3V, third ventricle. **b** Gene expression of *Gcn2* in amygdala (Amy) or arculate nucleus of the hypothalamus (ARC) by RT-PCR. **c** GCN2 proteins in Amy or ARC by western blotting (top) and quantified by densitometric analysis (bottom), A.U.: arbitrary units. **d** Immunofluorescence (IF) staining for GFP (green), GCN2 (red) and merge (yellow) in amygdala sections (n=3

per group). CeA: the central nuclei of the amygdala; BLA: the basolateral nuclei of the amygdala. **e** Gene expression of *ATF4, Trb3, Atf3* and *Gadd34* in amygdala by RT-PCR. **f** Epididymal WAT (eWAT) weight. **g** Representative images of hematoxylin and eosin (H&E) staining of eWAT. **h** eWAT cell size quantified by Image J analysis of H&E images. **i** Gene expression of *Ucp1, Pgc1a, Cidea, Dio2* and *Prdm16* in eWAT by RT-PCR. **j** Representative images of immunohistochemistry (IHC) of UCP1 in eWAT. **k** UCP1 protein in eWAT by western blotting (top) and quantified by densitometric analysis (bottom). All studies were conducted using 20- to 22-week-old male control mice ($GCN2^{+/+}$) or mice with GCN2 deletion in amygdala (GCN2 KO) fed a control (Control) or leucine-deficient [(-) L] diet for 3 days. Data are expressed as the mean ± SEM (n represents number of samples and are indicated above the bar graph), with individual data points. Data were analyzed by two-tailed unpaired Student's t test for **b** and **c**, or by one-way ANOVA followed by the SNK (Student–Newman–Keuls) test for **e**-**k**. Source data are provided as a Source Data file.

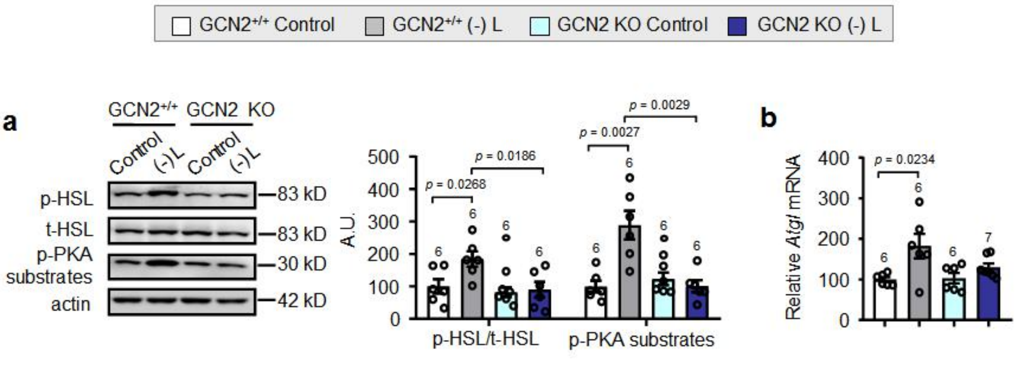

Supplementary Figure 7. The effect of GCN2 knockdown in amygdala on lipolysis-related gene and proteins in subcutaneous WAT (sWAT) under leucine

deprivation. a P-HSL, t-HSL and p-PKA substrates proteins in sWAT by western blotting (left) and quantified by densitometric analysis (right), A.U.: arbitrary units. **b** Gene expression of *Atgl* in sWAT by RT-PCR.All studies were conducted using 20- to 22-week-old male control mice (GCN2$^{+/+}$) or mice with GCN2 deletion in amygdala (GCN2 KO) fed a control (Control) or leucine-deficient [(-) L] diet for 3 days. Data are expressed as the mean ± SEM (n represents number of samples and are indicated above the bar graph), with individual data points. Data were analyzed by one-way ANOVA followed by the SNK (Student–Newman–Keuls) test. Source data are provided as a Source Data file.

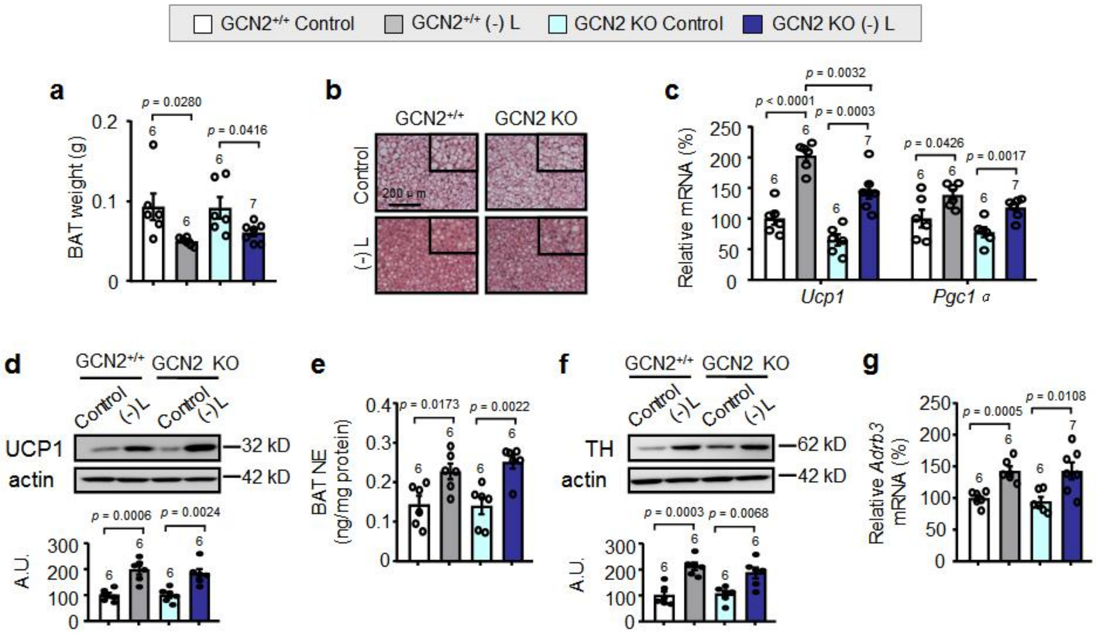

Supplementary Figure 8. GCN2 knockdown in amygdala has no obvious effect on BAT under leucine deprivation. a BAT weight. **b** Representative images of hematoxylin and eosin (H&E) staining of BAT (n=3 per group). **c**: Gene expression of *Ucp1* and *Pgc1a* in BAT by RT-PCR. **d** UCP1 protein in BAT by western blotting

(top) and quantified by densitometric analysis (bottom) , A.U.: arbitrary units. **e** Norepinephrine (NE) levels in BAT measured by ELISA kit. **f** TH protein in BAT by western blotting (top) and quantified by densitometric analysis (bottom). **g** Gene expression of *Adrb3* in BAT by RT-PCR. All studies were conducted using 20- to 22-week-old male control mice (GCN2$^{+/+}$) or mice with GCN2 deletion in amygdala (GCN2 KO) fed a control (Control) or leucine-deficient [(-) L] diet for 3 days. Data are expressed as the mean ± SEM (n represents number of samples and are indicated above the bar graph), with individual data points. Data were analyzed by one-way ANOVA followed by the SNK (Student–Newman–Keuls) test. Source data are provided as a Source Data file.

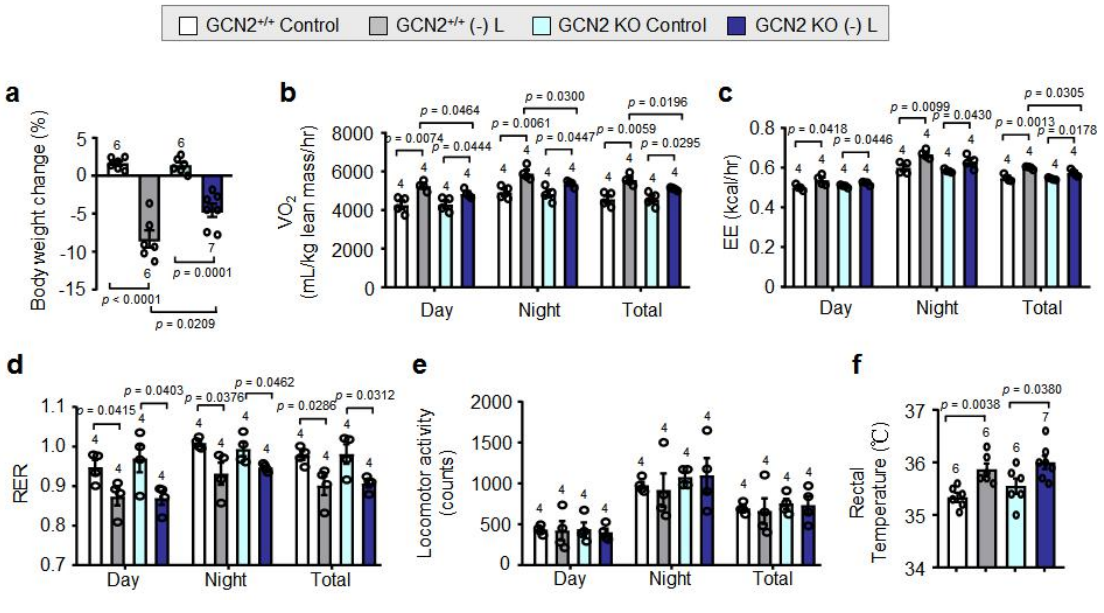

Supplementary Figure 9. The effect of GCN2 knockdown in amygdala on the indirect calorimetry and body temperature under leucine deprivation. a Body weight change relative to original body weight. **b** 24-h oxygen consumption normalized by lean mass measured by the comprehensive lab animal monitoring

system (CLAMS). **c** Energy expenditure (EE) measured by CLAMS. **d** Respiratory exchange ratio (RER, V_{CO2}/V_{O2}) measured by CLAMS. **e** Locomotor activity measured by CLAMS. **f** Rectal temperature by the digital thermometer.All studies were conducted using 20- to 22-week-old male control mice ($GCN2^{+/+}$) or mice with GCN2 deletion in amygdala (GCN2 KO) fed a control (Control) or leucine-deficient [(-) L] diet for 3 days. Data are expressed as the mean ± SEM (n represents number of samples and are indicated above the bar graph), with individual data points. Data were analyzed by one-way ANOVA followed by the SNK (Student–Newman–Keuls) test. Source data are provided as a Source Data file.

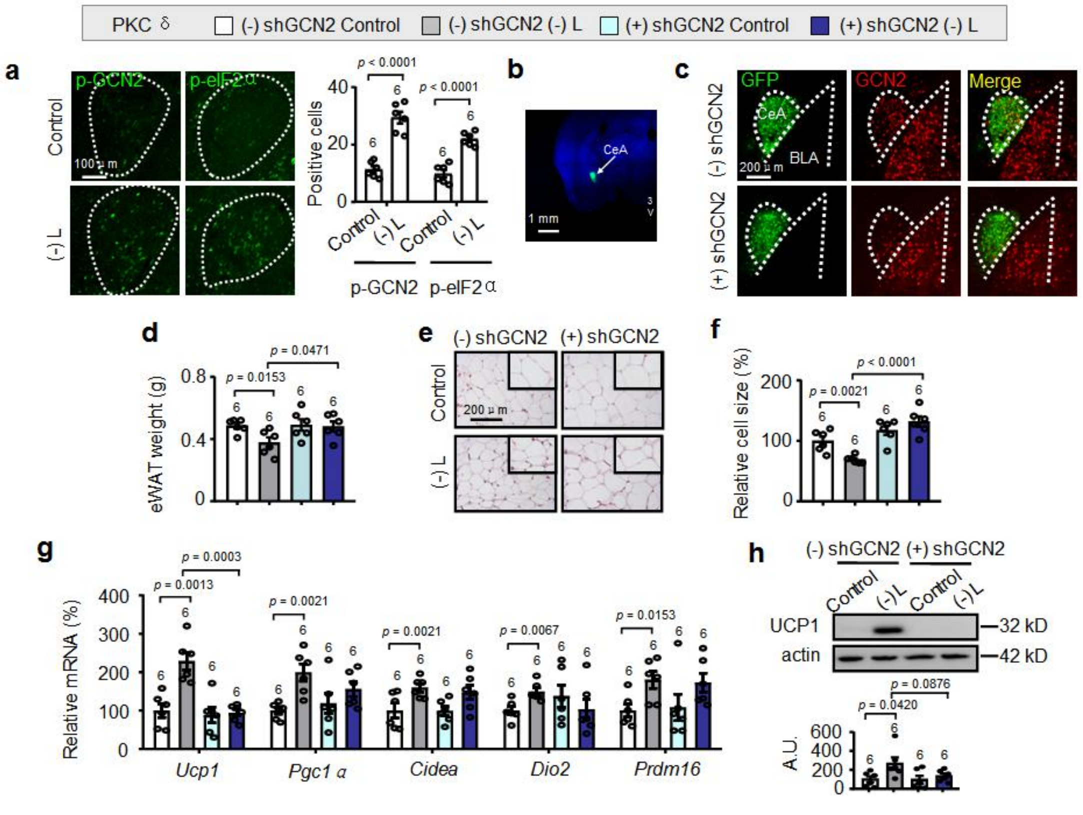

Supplementary Figure 10. Metabolic parameters related to mice with GCN2 knockdown in amygdalar PKC-δ neurons under leucine deprivation. a

Immunofluorescence (IF) staining for p-GCN2 and p-eIF2α (left) and quantification of p-GCN2 and p-eIF2α (right) in central amygdala. **b** Post hoc visualization of GFP (green) and DAPI (blue) in central amygdala (CeA) in PKC-δ-Cre mice receiving AAV-Flex-shGCN2-GFP stereotaxic injections (n = 3); 3V, third ventricle. **c** IF staining for GFP (green), GCN2 (red) and merge (yellow) in amygdala (n = 3 per group); CeA: the central nuclei of the amygdala; BLA: the basolateral nuclei of the amygdala. **d** Epididymal WAT (eWAT) weight. **e** Representative images of hematoxylin and eosin (H&E) staining of eWAT. **f** eWAT cell size quantified by Image J analysis of H&E images. **g** Gene expression of *Ucp1, Pgc1a, Cidea, Dio2* and *Prdm16* in eWAT by RT-PCR. **h** UCP1 protein in eWAT by western blotting (top) and quantified by densitometric analysis (bottom), A.U.: arbitrary units. Studies for **a** were conducted using 14- to 15-week-old male wild-type mice fed a control (Control) or leucine-deficient [(-) L] diet for 3 days; studies for **b**-**h** were conducted using 13- to 16-week-old male PKC-δ-Cre mice receiving AAVs expressing GFP (PKC δ - shGCN2) or shGCN2 (PKC δ + shGCN2) fed a Control or (-) L diet for 3 days. Data are expressed as the mean ± SEM (n represents number of samples and are indicated above the bar graph), with individual data points. Data were analyzed by two-tailed unpaired Student's t test for **a**, or one-way ANOVA followed by the SNK (Student–Newman–Keuls) test for **d-h**. Source data are provided as a Source Data file.

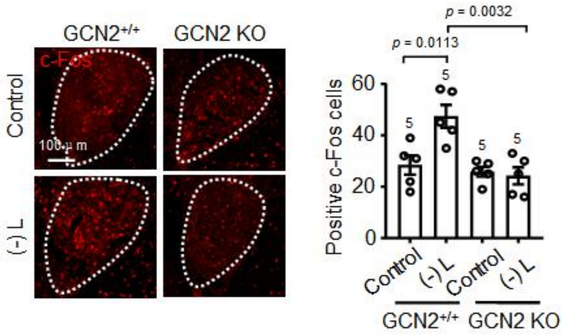

Supplementary Figure 11. C-Fos staining in the amygdala of amygdalar GCN2 deletion mice under leucine deprivation. Immunofluorescence (IF) staining for c-Fos (red) in central amygdala (CeA) sections (left) and quantification of c-Fos positive cells (right). Studies were conducted using 20- to 22-week-old male control mice (GCN2$^{+/+}$) or mice with GCN2 deletion in amygdala (GCN2 KO) fed a control (Control) or leucine-deficient [(-) L] diet for 3 days. Data are expressed as the mean ± SEM (n represents number of samples and are indicated above the bar graph), with individual data points. Data were analyzed by one-way ANOVA followed by the SNK (Student–Newman–Keuls) test. Source data are provided as a Source Data file.

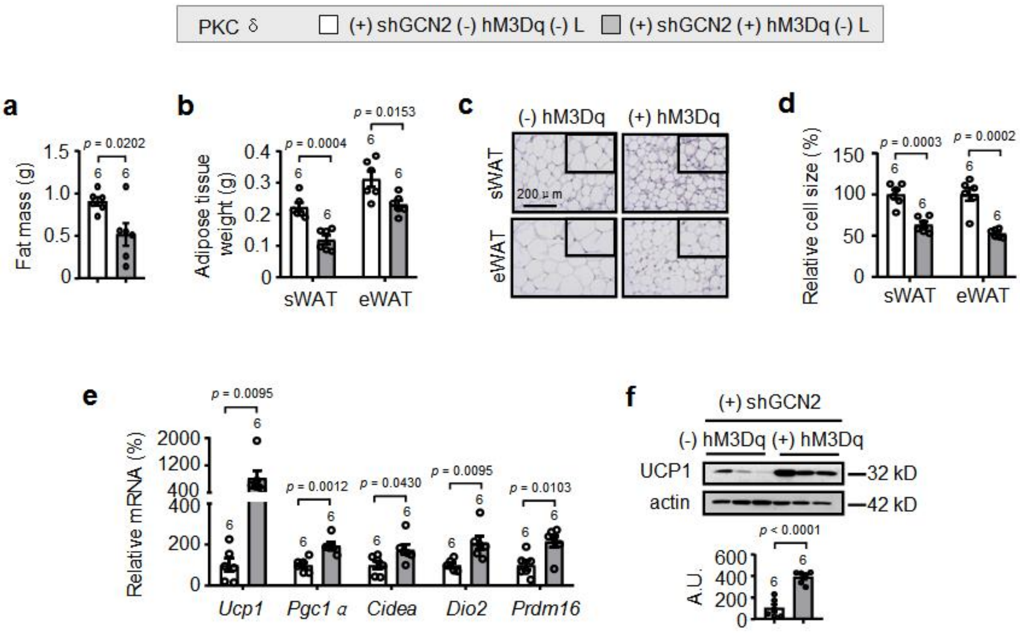

Supplementary Figure 12. Activation of PKC-δ neurons could reverse the blocking effect of GCN2 knockdown on leucine deprivation-induced browning. a Fat mass by NMR. **b** Subcutaneous WAT (sWAT) and epididymal WAT (eWAT) weight. **c** Representative images of hematoxylin and eosin (H&E) staining of sWAT and eWAT. **d** sWAT and eWAT cell size quantified by Image J analysis of H&E images. **e** Gene expression of *Ucp1, Pgc1a, Cidea, Dio2* and *Prdm16* in sWAT by RT-PCR. **f** UCP1 protein in sWAT by western blotting (top) and quantified by densitometric analysis (bottom), A.U.: arbitrary units. Studies were conducted using 13- to 15-week-old male PKC-δ-Cre mice receiving AAVs expressing shGCN2 and mCherry (PKCδ + shGCN2 - hM3Dq) or shGCN2 and hM3Dq (PKCδ + shGCN2 + hM3Dq), all received CNO injections every 12h for 3 days, simultaneously fed a leucine-deficient [(-) L] diet for 3 days. Data are expressed as the mean ± SEM (n represents number of samples and are indicated above the bar graph), with individual data points. Data were analyzed by two-tailed unpaired Student's t test. Source data

are provided as a Source Data file.

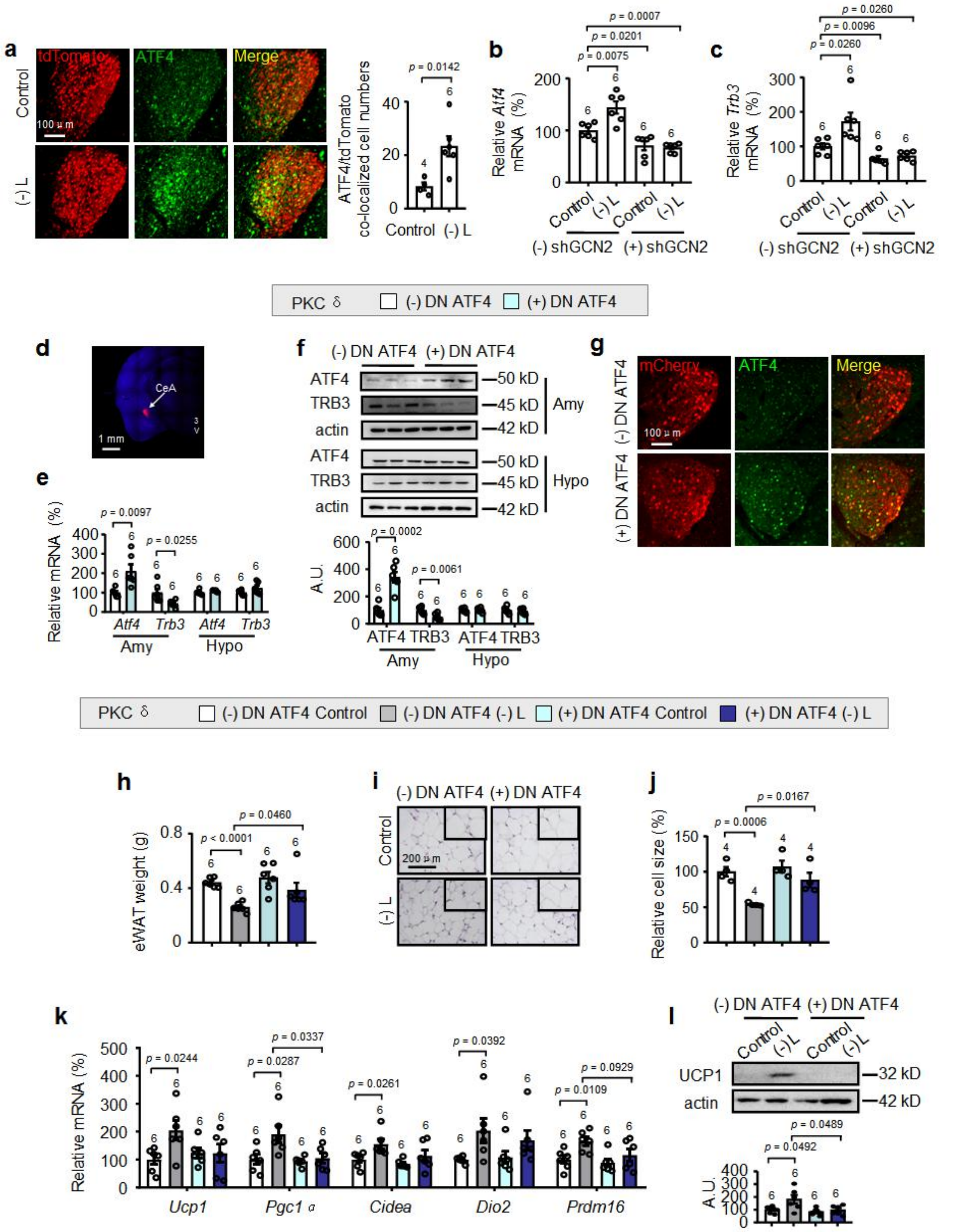

Supplementary Figure 13. Metabolic parameters related to mice with ATF4 knockdown in amygdalar PKC-δ neurons under leucine deprivation. a Immunofluorescence (IF) staining for tdTomato (red), ATF4 (green) and merge

(yellow) in central amygdala (CeA) sections (left), and quantification of ATF4 and tdTomato colocalized cell numbers (right). **b** Gene expression of *Atf4* in amygdala by RT-PCR. **c** Gene expression of *Trb3* in amygdala by RT-PCR. **d** Post hoc visualization of mCherry (red) and DAPI (blue) in CeA of PKC-δ-Cre mice receiving AAV-DIO-DN-ATF4-mCherry stereotaxic injections (n = 3); 3V, third ventricle. e Gene expression of *Atf4* and *Trb3* in amygdala (Amy) and hypothalamus (Hypo) by RT-PCR. **f** ATF4 and TRB3 proteins in Amy and Hypo by western blotting (left) and quantified by densitometric analysis (right), A.U.: arbitrary units. **g** IF staining for mCherry (red), ATF4 staining (green) or merge (yellow) in CeA sections (n = 3 per group). **h** Epididymal WAT (eWAT) weight. **i** Representative images of hematoxylin and eosin (H&E) staining of eWAT. **j** eWAT cell size quantified by Image J analysis of H&E images. **k** Gene expression of *Ucp1, Pgc1a, Cidea, Dio2* and *Prdm16* in eWAT by RT-PCR. **l** UCP1 protein in eWAT by western blotting (top) and quantified by densitometric analysis (bottom). Studies for **a** were conducted using 12- to 14-week-old male PKC-δ-Cre/Ai9 mice fed a control (Control) or leucine-deficient [(-) L] diet for 3 days; studies for **b** and **c** were conducted using 13- to 16-week-old male PKC-δ-Cre mice receiving AAVs expressing GFP (PKC δ - shGCN2) or shGCN2 (PKC δ + shGCN2) fed a Control or (-) L diet for 3 days; studies for **d**-**l** were conducted using or 13- to 16-week-old male PKC-δ-Cre mice receiving AAVs expressing mCherry (PKCδ - DN ATF4) or DN ATF4 (PKCδ + DN ATF4) fed a Control or (-) L diet for 3 days. Data are expressed as the mean ± SEM (n represents number of samples and are indicated above the bar graph), with individual data points.

Data were analyzed by two-tailed unpaired Student's t test for **a**, **d** and **f**, or by one-way ANOVA followed by the SNK (Student–Newman–Keuls) test for **b**, **c** and **h**-**l**. Source data are provided as a Source Data file.

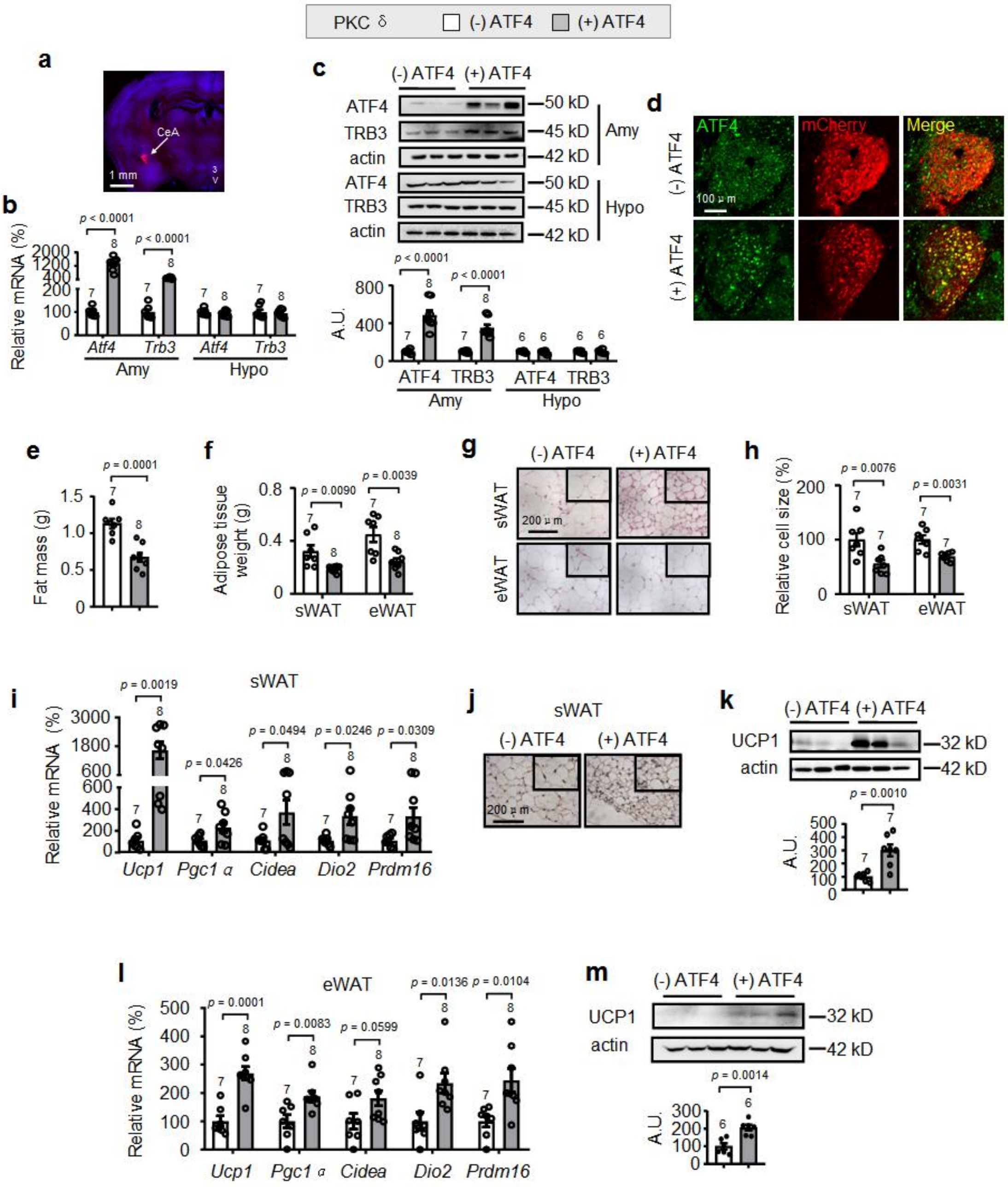

Supplementary Figure 14. Over-expression of ATF4 in amygdalar PKC-δ neurons mimics leucine deprivation-induced WAT browning. a Post hoc

visualization of mCherry (red) and DAPI (blue) in central amygdala (CeA) of PKC-δ-Cre mice receiving AAV-DIO-ATF4-GFP stereotaxic injections (n = 3); 3V, third ventricle. **b** Gene expression of *Atf4* and *Trb3* in amygdala (Amy) and hypothalamus (Hypo). **c** ATF4 and TRB3 proteins in Amy and Hypo by western blotting (left) and quantified by densitometric analysis (right), A.U.: arbitrary units. **d** Immunofluorescence (IF) staining for mCherry (red), ATF4 (green) or merge (yellow) in CeA (n = 3 per group). **e** Fat mass by NMR. **f** Adipose tissue weight. **g** Representative images of hematoxylin and eosin (H&E) staining of subcutaneous WAT (sWAT) and epididymal WAT (eWAT). **h** sWAT and eWAT cell size quantified by Image J analysis of H&E images. **i** Gene expression of *Ucp1, Pgc1a, Cidea, Dio2* and *Prdm16* in sWAT by RT-PCR. **j** Representative images of immunohistochemistry (IHC) of UCP1 in sWAT. **k** UCP1 protein in sWAT by western blotting (top) and quantified by densitometric analysis (bottom). **l** Gene expression of *Ucp1, Pgc1a, Cidea, Dio2* and *Prdm16* in eWAT by RT-PCR**.** **m** UCP1 protein in eWAT by western blotting (top) and quantified by densitometric analysis (bottom). Studies were conducted using 13- to 15-week-old male PKC-δ-Cre mice receiving AAVs expressing mCherry (PKC δ - ATF4) or ATF4 (PKC δ + ATF4). Data are expressed as the mean ± SEM (n represents number of samples and are indicated above the bar graph), with individual data points. Data were analyzed by two-tailed unpaired Student's t test. Source data are provided as a Source Data file.

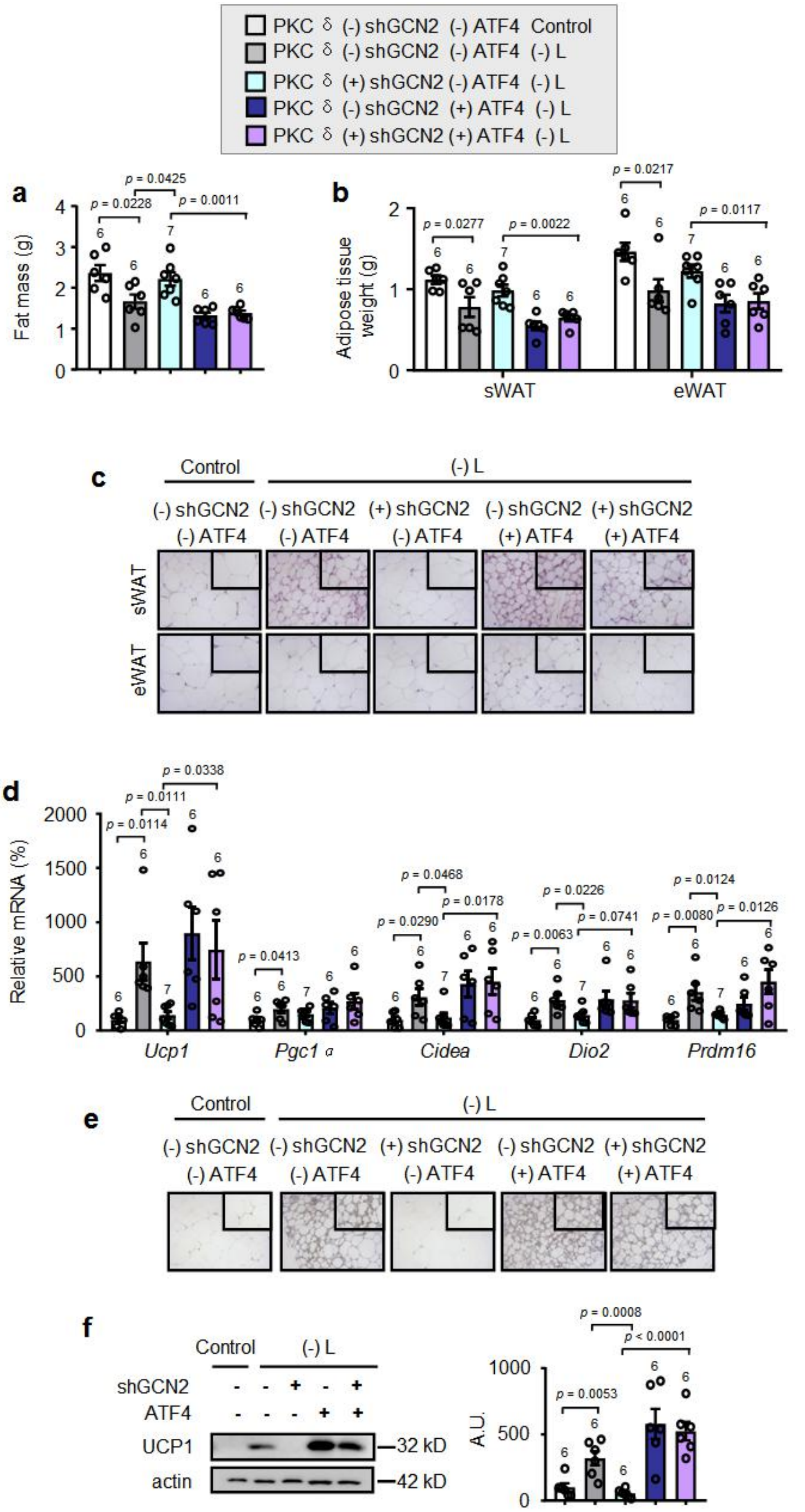

Supplementary Figure 15. Metabolic parameters related to mice with GCN2 knockdown with or without ATF4 over-expression in amygdalar PKC-δ neurons under leucine deprivation. a Fat mass by NMR. **b** Adipose tissue weight. **c**

Representative images of hematoxylin and eosin (H&E) staining of subcutaneous WAT (sWAT) and epididymal WAT (eWAT)(n = 3 per group). **d** Gene expression of *Ucp1, Pgc1a, Cidea, Dio2* and *Prdm16* in sWAT by RT-PCR. **e** Representative images of immunohistochemistry (IHC) of UCP1 in sWAT. **f** UCP1 protein in sWAT by western blotting (left) and quantified by densitometric analysis (right), A.U.: arbitrary units. Studies were conducted using 20- to 22-week-old male PKC-δ-Cre mice receiving AAVs expressing GFP and mCherry (PKC δ - shGCN2 - ATF4), or shGCN2 and mCherry (PKC δ + shGCN2 - ATF4), or GFP and ATF4 (PKC δ - shGCN2 + ATF4), or shGCN2 and ATF4 (PKC δ + shGCN2 + ATF4) fed a leucine-deficient [(-) Leu] diet for 3 days; or receiving AAVs expressing GFP and mCherry (PKC δ - shGCN2 - ATF4) fed a control diet (Control) for 3 days. Data are expressed as the mean ± SEM (n represents number of samples and are indicated above the bar graph), with individual data points. Data were analyzed by one-way ANOVA followed by the SNK (Student–Newman–Keuls) test. Source data are provided as a Source Data file.

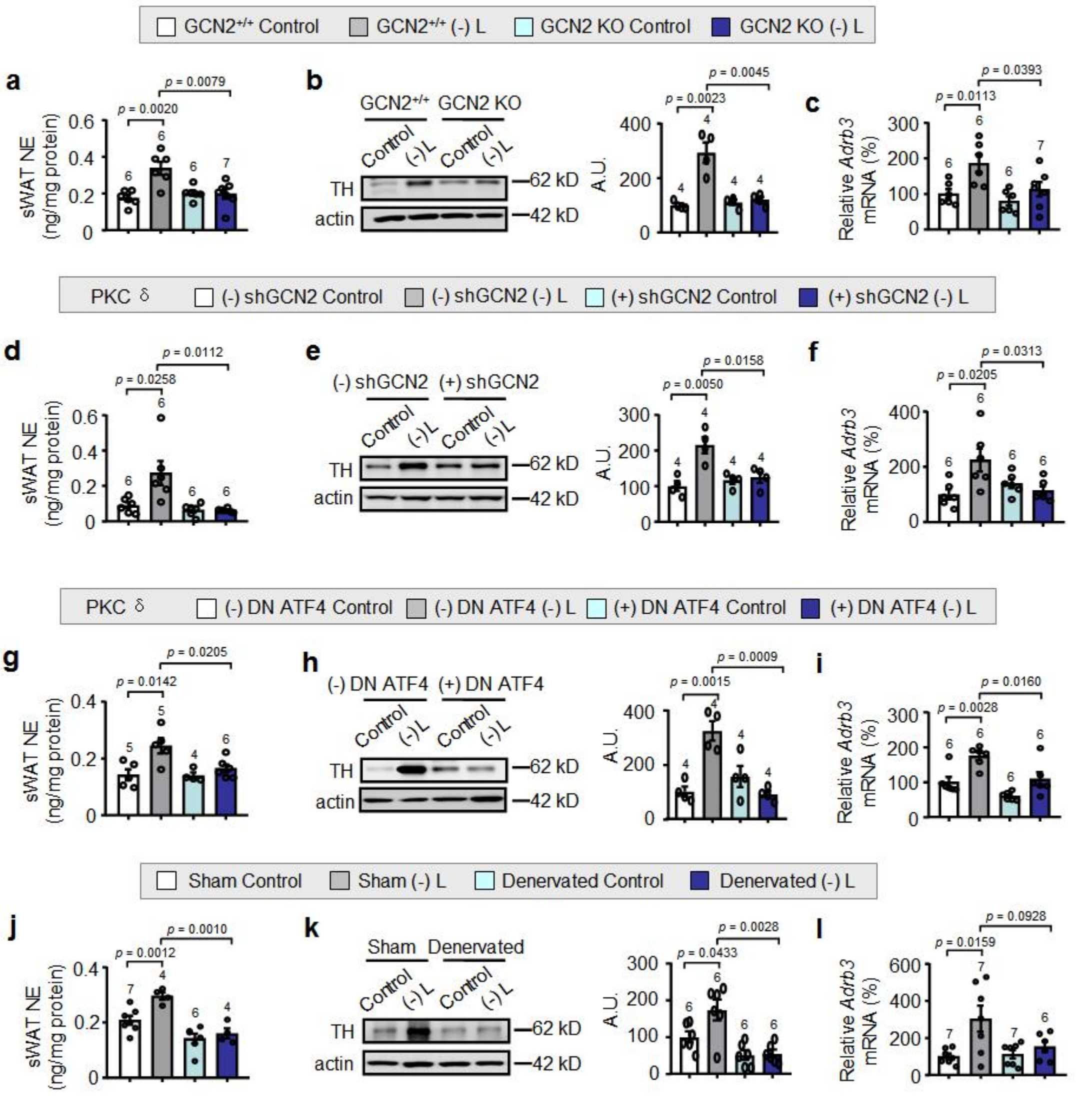

Supplementary Figure 16. The activity of sympathetic nervous system (SNS) in several groups of mice. a Norepinephrine (NE) levels in subcutaneous WAT (sWAT) measured by ELISA kit. **b** Tyrosine hydroxylase (TH) protein in sWAT by western blotting (top) and quantified by densitometric analysis (bottom), A.U.: arbitrary units. **c** Gene expression of *Adrb3* in sWAT by RT-PCR. **d** NE levels in sWAT measured by ELISA kit. **e** TH protein in sWAT by western blotting (top) and quantified by densitometric analysis (bottom). **f** Gene expression of *Adrb3* in sWAT by RT-PCR. **g** NE levels in sWAT measured by ELISA kit. **h** TH protein in sWAT by western blotting (top) and quantified by densitometric analysis (bottom). **i** Gene expression of

Adrb3 in sWAT by RT-PCR. **j** NE levels in sWAT measured by ELISA kit. **k** TH protein in sWAT by western blotting (top) and quantified by densitometric analysis (bottom). **l** Gene expression of *Adrb3* in sWAT by RT-PCR. Studies for **a-c** were conducted using 20- to 22-week-old male control mice (GCN2$^{+/+}$) or mice with GCN2 deletion in amygdala (GCN2 KO) fed a control (Control) or leucine-deficient [(-) L] diet for 3 days; studies for **d-f** were conducted using 13- to 16-week-old male PKC-δ-Cre mice receiving AAVs expressing GFP (PKC δ - shGCN2) or shGCN2 (PKC δ + shGCN2) fed a Control or (-) L diet for 3 days; studies for **g-i** were conducted using 13- to 16-week-old male PKC-δ-Cre mice receiving AAVs expressing GFP (PKC δ - DN ATF4) or DN ATF4 (PKC δ + DN ATF4) fed a Control or (-) L diet for 3 days; studies for **j-l** were conducted using 12-week-old male WT mice with sham operated (Sham) or denervated (Denervated) fed a Control or (-) L diet for 3 days. Data are expressed as the mean ± SEM (n represents number of samples and are indicated above the bar graph), with individual data points. Data were analyzed by one-way ANOVA followed by the SNK (Student–Newman–Keuls) test. Source data are provided as a Source Data file.

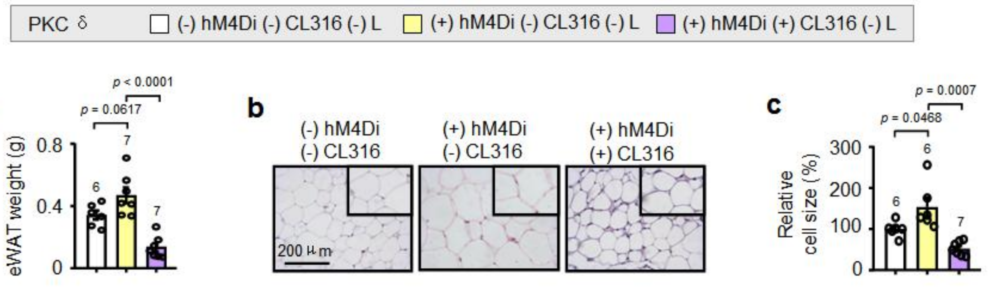

Supplementary Figure 17. Metabolic parameters related to mice with inhibition

of PKC-δ neurons' activity and treated with CL316 under leucine deprivation. a Epididymal WAT (eWAT) weight. **b.** Representative images of hematoxylin and eosin (H&E) staining of eWAT. **c** eWAT cell size quantified by Image J analysis of H&E images. Studies were conducted using 16- to 18-week-old male PKC δ - hM4Di or PKC δ + hM4Di mice injected with saline (- CL316) or CL316243 (+ CL316), all received CNO injections every 12h for 3 days, fed a leucine-deficient [(-) L] diet for 3 days. Data are expressed as the mean ± SEM (n represents number of samples and are indicated above the bar graph), with individual data points. Data were analyzed by one-way ANOVA followed by the SNK (Student–Newman–Keuls) test. Source data are provided as a Source Data file.

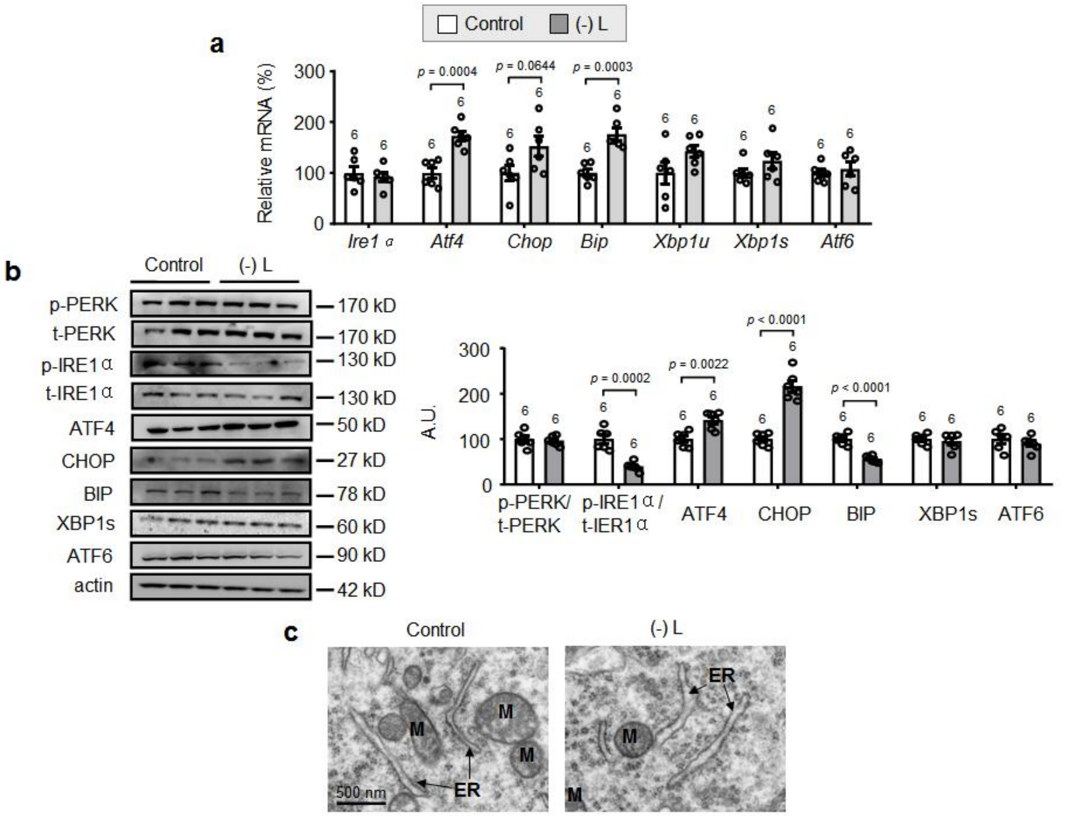

Supplementary Figure 18. The signals of ER stress in amygdala under leucine

deprivation. a Gene expression of *Ire1a*, *Atf4*, *Chop*, *Bip*, *Xbp1u*, *Xbp1s* and *Atf6* by RT-PCR. **b** P-PERK, t-PERK, p-IRE1α, t-IRE1α, ATF4, CHOP, BIP, XBP1s and ATF6 proteins by western blotting (left) and quantified by densitometric analysis (right), A.U.: arbitrary units. **c** Electron microscopy (EM) analysis of the amygdala (n = 3 per group); ER: endoplasmic reticulum; M: mitochondria. Studies were conducted using 14- to 15-week-old male wild-type mice fed a control (Control) or leucine-deficient [(-) L] diet for 3 days. Data are expressed as the mean ± SEM (n represents number of samples and are indicated above the bar graph), with individual data points. Data were analyzed by two-tailed unpaired Student's t test. Source data are provided as a Source Data file.

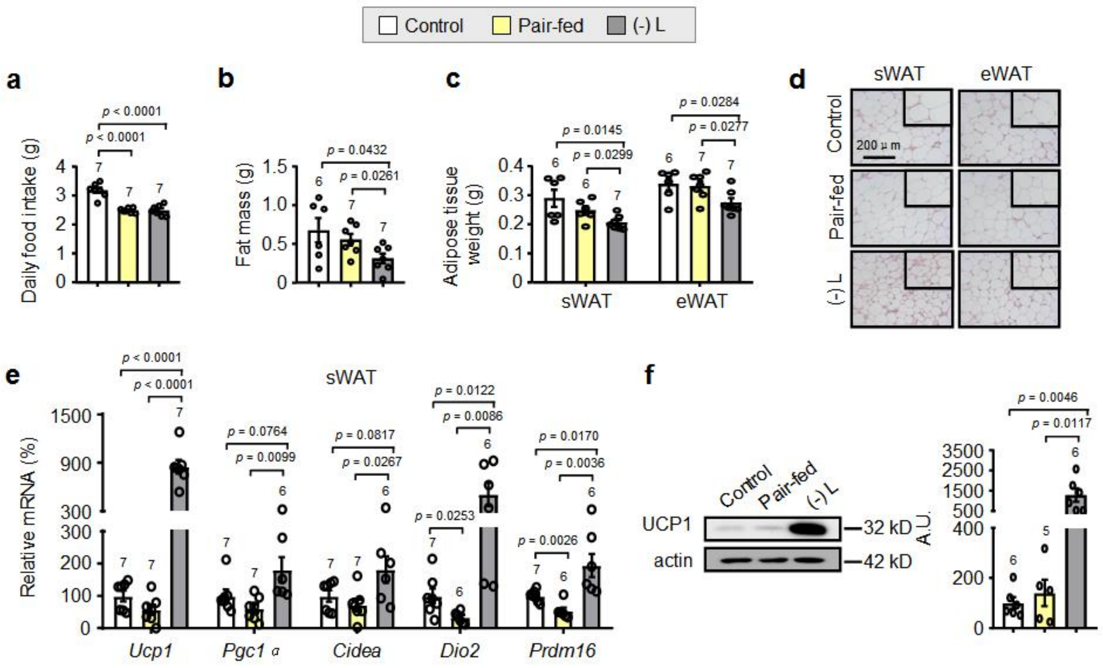

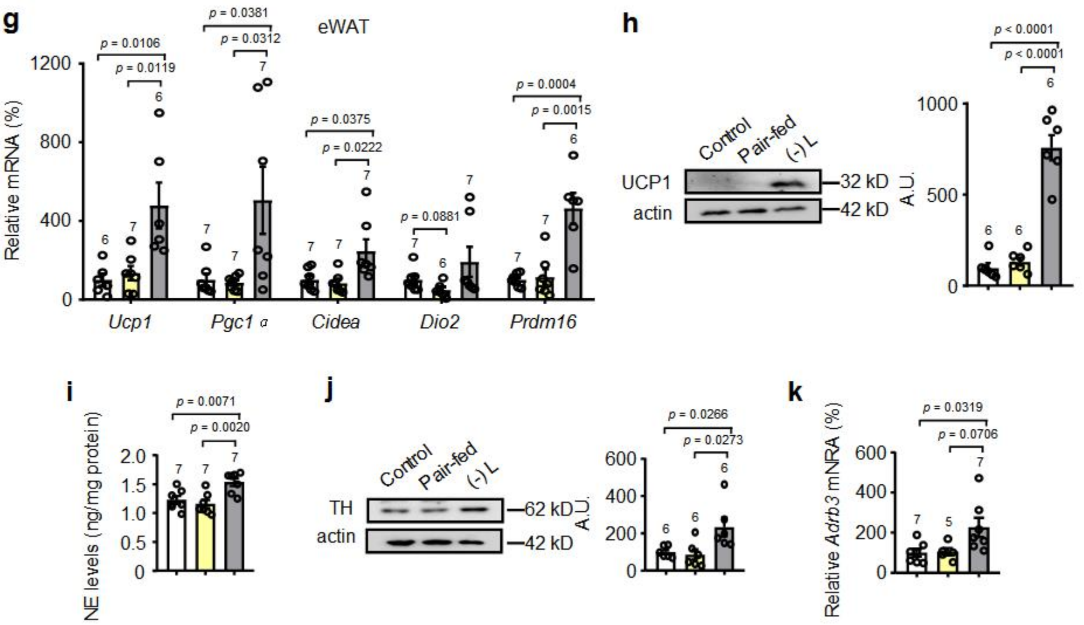

Supplementary Figure 19. Pair-feeding has no significant impact on WAT browning and sympathetic nervous system activity. a Daily food intake. **b** Fat mass by NMR. **c** Subcutaneous WAT (sWAT) and epididymal WAT (eWAT) weight. **d** Representative images of hematoxylin and eosin (H&E) staining of sWAT and eWAT (n = 3 per group). **e** Gene expression of *Ucp1, Pgc1a, Cidea, Dio2* and *Prdm16* in sWAT by RT-PCR. **f** UCP1 protein in sWAT by western blotting (left) and quantified by densitometric analysis (right), A.U.: arbitrary units. **g** Gene expression of *Ucp1, Pgc1a, Cidea, Dio2* and *Prdm16* in eWAT by RT-PCR. **h** UCP1 protein in eWAT by western blotting (left) and quantified by densitometric analysis (right). **i** Norepinephrine (NE) levels in sWAT measured by ELISA kit. **j** TH protein in sWAT by western blotting (left) and quantified by densitometric analysis (right). **k** Gene expression of *Adrb3* in sWAT by RT-PCR. Studies were conducted using 8-week-old male WT mice fed a control (Control), leucine-deficient [(-) L], or pair-fed (Pair-fed) diet for 3 days. Data are expressed as the mean ± SEM (n

represents number of samples and are indicated above the bar graph), with individual data points. Data were analyzed by one-way ANOVA followed by the SNK (Student–Newman–Keuls) test. Source data are provided as a Source Data file.

Supplementary Table 1. Primers used for gene amplification.

**Gene**	**Direction**	**Primer sequence 5'→3'**
GCN2	F<br>R	CCTGCACCATGAGAACATTG<br>CTGCCCAGTTCTTCAGTGT
UCP1	F<br>R	ACTGCCACACCTCCAGTCATT<br>CTTTGCCTCACTCAGGATTGG
PGC1α	F<br>R	GATGGCACGCAGCCCTAT<br>CTCGACACGGAGAGTTAAAGGAA
CIDEA	F<br>R	TGCTCTTCTGTATCGCCCAGT<br>GCCGTGTTAAGGAATCTGCTG
DIO2	F<br>R	TGTGGTGCACGTCTCCAATC<br>GCCCCATCAGCGGTCTT
PRDM16	F<br>R	CAGCACGGTGAAGCCATTC<br>GCGTGCATCCGCTTGTG
ADRB3	F<br>R	ACGCCGAGACTACAGACCATA<br>CTGGTGGCATTACGAGGA
ATF4	F<br>R	CCTGAACAGCGAAGTGTTGG<br>TGGAGAACCCATGAGGTTTCAA
TRB3	F<br>R	TGTCTTGCGCGACCTCAA<br>CCAGCTTCGTCCTCTCACAGT
GAPDH	F<br>R	TGTGTCCGTCGTGGATCTGA<br>CCTGCTTCACCACCTTCTTGAT
ATF3	F<br>R	GAGGATTTTGCTAACCTGACACC<br>TTGACGGTAACTGACTCCAGC
GADD34	F<br>R	AGGACCCCGAGATTCCTCTA<br>AGGTAGGGACCCAGCTTCTC
ATGL	F<br>R	GTGAAGCAGGTGCCAACATTATTG<br>AAACACGAGTCAGGGAGATGCC
IRE1α	F<br>R	CGCACATGGCAGGATCAGG<br>TGCCCACTGCCAGCTTCT
CHOP	F<br>R	GGGCCAACAGAGGTCACAC<br>CTTCATGCGTTGCTTCCCA
BIP	F	ACTTGGGGACCACCTATTCCT

	R	ATCGCCAATCAGACGCTCC
XBP1u	F	AGCAGCAAGTGGTGGATTTG
	R	GAGTTTTCTCCCGTAAAAGCTGA
XBP1s	F	ACACGCTTGGGAATGGACAC
	R	CCATGGGAAGATGTTCTGGG
ATF6	F	CGGTCCACAGACTCGTGTTC
	R	GCTGTCGCCATATAAGGAAAGG
