# Supplementary material for: Activation of GCN2/ATF4 signals in amygdalar PKC-δ neurons promotes WAT browning under leucine deprivation
Source: Nat Commun. 2020 Jun 5;11:2847. doi: 10.1038/s41467-020-16662-2 (PMC7275074; doi:10.1038/s41467-020-16662-2)
Supplement: Supplementary file 3 — Reporting Summary [file 41467_2020_16662_MOESM3_ESM.pdf]

nature research

Corresponding author(s): Feifan Guo

Last updated by author(s): Apr 14, 2020

Reporting Summary

Nature Research wishes to improve the reproducibility of the work that we publish. This form provides structure for consistency and transparency in reporting. For further information on Nature Research policies, see Authors & Referees and the Editorial Policy Checklist .

Statistics

For all statistical analyses, confirm that the following items are present in the figure legend, table legend, main text, or Methods section.

n/a	Confirmed	
☐	☒	The exact sample size (*n*) for each experimental group/condition, given as a discrete number and unit of measurement
☐	☒	A statement on whether measurements were taken from distinct samples or whether the same sample was measured repeatedly
☐	☒	The statistical test(s) used AND whether they are one- or two-sided<br>*Only common tests should be described solely by name; describe more complex techniques in the Methods section.*
☒	☐	A description of all covariates tested
☒	☐	A description of any assumptions or corrections, such as tests of normality and adjustment for multiple comparisons
☐	☒	A full description of the statistical parameters including central tendency (e.g. means) or other basic estimates (e.g. regression coefficient) AND variation (e.g. standard deviation) or associated estimates of uncertainty (e.g. confidence intervals)
☐	☒	For null hypothesis testing, the test statistic (e.g. *F*, *t*, *r*) with confidence intervals, effect sizes, degrees of freedom and *P* value noted<br>*Give P values as exact values whenever suitable.*
☒	☐	For Bayesian analysis, information on the choice of priors and Markov chain Monte Carlo settings
☒	☐	For hierarchical and complex designs, identification of the appropriate level for tests and full reporting of outcomes
☒	☐	Estimates of effect sizes (e.g. Cohen's *d*, Pearson's *r*), indicating how they were calculated

Our web collection on statistics for biologists contains articles on many of the points above.

Software and code

Policy information about availability of computer code

Data collection	Fluorescent images were collected using Zeiss LSM780 confocal microscopy; Western Blot images were collected with Tanon MP software; UCP1 IHC images and H&E images were collected using QCapture microscope ; the body fat mass and lean mass were collected using nuclear magnetic resonance system (Bruker, DE, USA); the oxygen consumption, energy expenditure, respiratory exchange ratio and locomotor activity were collected using the comprehensine lab animal monitoring system (CLAMS; Columbus Instruments, OH, USA); the body temperature was collected using a rectal probe attached to a digital thermometer (Physitemp Instruments, NJ, USA); the RT-PCR data were collected using ABI 7900 system; the electron microscopy images were collected using the JEOL transmission electron microscopy.
Data analysis	Fluorescent images were processed in ZEN and Photoshop CS5; Graph PadPrism 8 and Microsoft Office Excel were used for statistic analysis; Serum and amygdalar amino acids were analyzed with high performance liquid chromatography (Ultimate 3000, USA) tandem mass spectrometry (API 3200 Q-TRAP, USA); the Western Blot images were analyzed using Tanon Gis.

For manuscripts utilizing custom algorithms or software that are central to the research but not yet described in published literature, software must be made available to editors/reviewers. We strongly encourage code deposition in a community repository (e.g. GitHub). See the Nature Research guidelines for submitting code & software for further information.

Data

Policy information about availability of data

All manuscripts must include a data availability statement. This statement should provide the following information, where applicable:

- Accession codes, unique identifiers, or web links for publicly available datasets
- A list of figures that have associated raw data
- A description of any restrictions on data availability

The authors declare that all data presented in this study are available within the Figures and its Supplementary Information file. The source data underlying Figures 1-7 and Supplementary Figures S1-S19 are provided as a Source Data file. Other data that support the study are available from the corresponding author upon reasonable request.

Field-specific reporting

Please select the one below that is the best fit for your research. If you are not sure, read the appropriate sections before making your selection.

[x] Life sciences [ ] Behavioural & social sciences [ ] Ecological, evolutionary & environmental sciences

For a reference copy of the document with all sections, see nature.com/documents/nr-reporting-summary-flat.pdf

Life sciences study design

All studies must disclose on these points even when the disclosure is negative.

Sample size	No sample size calculation was performed, but sample sizes were sufficient to carry out the required experiments with sufficient statistics and standard using t-test for such kind of experiments. In addition, each figure legend describes the number of technical and biological replicates.
Data exclusions	Western Blot analysis in supplementary figure 1f excluded the minimum and the maximum data to fit in the requirement of the journal for n ≤ 10 per group. No other data were excluded.
Replication	The data reported were generated using at least three different biological replicate in most required experiments.
Randomization	Mice were allocated randomly.
Blinding	The investigators were not blinded to allocation during experiments and data analysis, as objective quantitative assays were used when we generated the data.

Reporting for specific materials, systems and methods

We require information from authors about some types of materials, experimental systems and methods used in many studies. Here, indicate whether each material, system or method listed is relevant to your study. If you are not sure if a list item applies to your research, read the appropriate section before selecting a response.

Materials & experimental systems

n/a	Involved in the study	
[ ]	[x]	Antibodies
[x]	[ ]	Eukaryotic cell lines
[x]	[ ]	Palaeontology
[ ]	[x]	Animals and other organisms
[x]	[ ]	Human research participants
[x]	[ ]	Clinical data

Methods

n/a	Involved in the study	
[x]	[ ]	ChIP-seq
[x]	[ ]	Flow cytometry
[x]	[ ]	MRI-based neuroimaging

Antibodies

Antibodies used	anti-p-GCN2 (Biorbyt, orb6078); anti-GCN2 (Cell Signaling,#3302) for western blots, anti-GCN2 (Abcam,ab137543) for immunofluorescence; anti-UCP1 (Abcam,Ab10983); anti-β-actin(Sigma Aldrich,A5316);anti-p-EIF2α(Cell Signaling,#3398); anti-EIF2α (Cell Signaling,#9722); anti-TRB3(Santa cruz,sc-34211); anti-ATF4(Santa cruz,sc-200); anti-c-Fos(Santa cruz, sc-52); anti-TH (Merck, AB152), anti-p-PERK (SAB, 12814-1), anti-PERK (Proteintech, 20582-1-AP), anti-p-IRE1α (Epitomics, 3881-1),.anti-IRE1α (Cell Signaling, #3294), anti-p-HSL (Cell Signaling,#4126); anti-HSL(Cell Signaling,#4107); anti-p-PKA substrates (Cell Signaling,#9621); anti-CHOP (Proteintech, 15204-1-AP), anti-BIP (Sigma, G8918), anti-XBP1s (Proteintech, 24868-1-AP) and anti-ATF6 (Abcam, ab37149).
Validation	All antibodies used in this work were purchased from companies, and validated by the manufacturers and by extensive use in

	published work.

Animals and other organisms

Policy information about studies involving animals; ARRIVE guidelines recommended for reporting animal research

Laboratory animals	The mice used in this study were included in the method and figure legend section of the manuscript. Wild type C57BL/6J mice, PKC-δ-Cre mice, Ai9 (tdTomato) reporter mice, global GCN2 knockout mice and GCN2-floxed mice were used. All the mice used in the study were male, aged 8-22-week old. Mice were housed according to a 12-h light (7 a.m.)/dark (7 p.m.) cycle at 25 ℃, at a constant relative humidity of 45%, with ad libitum access to water and rodent standard chow diet prior to the experiments.
Wild animals	The study did not involve wild animals.
Field-collected samples	The study did not involve samples collected from the field.
Ethics oversight	The experiments were conducted in accordance with guidelines of the Institutional Animal Care and Use Committee of the Shanghai Institute of Nutrition and Health, Shanghai Institutes for Biological Sciences, Chinese Academy of Sciences.

Note that full information on the approval of the study protocol must also be provided in the manuscript.
